# Case report: Durable response of ensartinib targeting EML4-ALK fusion in osimertinib-resistant non-small cell lung cancer

**DOI:** 10.3389/fphar.2024.1359403

**Published:** 2024-07-29

**Authors:** Yongkuan Guo, Ran Zhang, Yiran Meng, Li Wang, Liuqing Zheng, Jian You

**Affiliations:** ^1^ Department of Thoracic Oncology, Tianjin Cancer Hospital Airport Hospital, National Clinical Research Center for Cancer, Tianjin, China; ^2^ Hangzhou Repugene Technology Co., Ltd., Hangzhou, China; ^3^ Department of Pulmonary Oncology, Tianjin Medical University Cancer Institute and Hospital, National Clinical Research Center for Cancer, Tianjin's Clinical Research Center for Cancer, Tianjin, China

**Keywords:** ensartinib, EML4-ALK, osimertinib, resistance mechanism, case report

## Abstract

**Background:**

Despite significant benefits from targeted therapy in patients with driver mutations, inevitable drug resistance usually occurred in non-small cell lung cancer, highlighting the necessity for sequential treatments to prolong overall survival. Unfortunately, durable drug response has not been reported in posterior-line therapy of cases with acquired *EML4-ALK* fusion after resistance to osimertinib, urging the need of referable decision-making in clinical management.

**Case presentation:**

We present a case of a 71-year-old Chinese female, never smoker, diagnosed with invasive adenocarcinoma in the left inferior lobe of her lung, with metastases in regional lymph nodes. She received erlotinib treatment after the detection of coexistent *EGFR* L858R/G719S and *BRAF* V600E via next-generation sequencing of resected tumor tissue. Routine imaging revealed disease progression approximately 14 months after starting erlotinib treatment, followed by the detection of *EGFR* L858R through non-invasive liquid biopsy. Subsequently, osimertinib was administered, showing clinical activities for nearly 19 months until the emergence of an *EML4-ALK* fusion. Given the *EML4-ALK* fusion, a relatively rare resistance mechanism to osimertinib, she received third-line ensartinib treatment. One month later, alleviated tumor lesions plus normal serum marker levels demonstrated the effectiveness of ensartinib in overcoming resistance to osimertinib. Of note, the clinical response to ensartinib persisted for more than 14 months, superior to the previously reported efficacy of aletinib and crizotinib in osimertinib-failure cases. As of the last follow-up in July 2022, the patient showed no signs of recurrence and maintained a good life quality.

**Conclusion:**

We reported a third-line ensartinib therapy in a patient with lung adenocarcinoma who developed an acquired *EML4-ALK* fusion after sequential treatment with erlotinib and osimertinib. Given the rarity of the *EML4-ALK* fusion as a resistance mechanism to osimertinib, ensartinib emerges as a promising treatment option for this specific clinical challenge, offering superior efficacy and good safety.

## 1 Introduction

Non-small cell lung cancer (NSCLC) accounts for 80%–85% of lung cancer and is the leading cause of cancer-related death worldwide (Alexander et al., 2020). Mutations in the epidermal growth factor receptor (*EGFR*) gene are significant targets in NSCLC, affecting nearly 50% of East-Asian carriers. Three generations of EGFR tyrosine kinase inhibitors (TKIs) have been developed to target different sensitive mutant sites. The first-generation reversible EGFR-TKIs, such as gefitinib, erlotinib, and icotinib, are the primary choices of first-line treatment, offering significant survival benefits and good drug safety (Gazdar, 2009; Huang et al., 2020). Due to their high incidence of adverse effects, the clinical application of second-generation EGFR-TKIs, including afatinib and dacomitinib, is relatively limited (Huang et al., 2020). Osimertinib, a third-generation EGFR-TKI, exhibits favorable safety and excellent central nervous system (CNS) penetration and is recommended as the new first-line therapy of advanced NSCLC patients in routine practice (Leonetti et al., 2019; Huang et al., 2020).

However, development of resistance is an inevitable challenge in TKI therapy. The acquired resistance mechanisms to osimertinib can be classified into two major types: EGFR-dependent mechanisms, such as *EGFR* T790M mutation, and EGFR-independent mechanisms, predominately characterized by *MET/HER2* amplification and other bypass activated mutations (Leonetti et al., 2019). In a relatively small proportion of osimertinib-resistant patients, acquired *EML4-ALK* fusion has been detected (Ho et al., 2017; Minari et al., 2018; Offin et al., 2018). This resistance mechanism has also been identified in patients treated with first- and second-generation TKIs. In an early-stage NSCLC patient with treatment history of adjuvant chemotherapy plus gefitinib, dual mutations of *ALK-R3HDM1* (A19: R21) and *EML4-ALK* (E6: A20, variant 3) rearrangement were detected in his metastatic lymph node. A case that received second-line erlotinib and chemotherapy developed acquired *EML4-ALK* fusion and *EGFR* 19del mutations (Zeng et al., 2022). *EML4-ALK* fusion has also been reported to serve as the resistant mechanism to afatinib in an 80-year-old male (Xu et al., 2019). A review enrolling 24 NSCLC cases reported that three of seven afatinib-relapsed patients harbored positive *EML4-ALK* fusion (Schrock et al., 2018). For third generation EGFR-TKIs, in addition to osimertinib, acquired *EML4-ALK* fusion was also identified in an almonertinib-relapsed NSCLC (Ren et al., 2022). Whether influenced by the extensive application in clinical practice or TKI generations, patients developing acquired *EML4-ALK* fusion constitute a significant portion of osimertinib-replased population (Passaro et al., 2021; Zeng et al., 2022).

The *EML4-ALK* rearrangement is another oncogenic driver mutation that occurs in 3%–5% of NSCLC and can be targeted by ALK-TKIs (McCusker et al., 2019). Crizotinib, a first-generation ALK-TKI, shows superiority over chemotherapy for *ALK*-rearranged NSCLC but has limited effects in patients with CNS involvement. This limitation has been significantly improved by the second-generation ALK-TKIs, including ceritinib, alectinib, brigatinib, and ensartinib. Additionally, lorlatinib, a third-generation ALK-TKI, has been developed to overcome the primary and acquired resistance to earlier-generation ALK inhibitors (Wu et al., 2016). Prior to the demonstration of the safety and superior efficacy of second- and third-generation ALK-TKIs in clinical trials, crizotinib was the primary first-line therapeutic strategy for treatment-naive patients. Consequently, in previously reported cases of osimertinib-resistant patients developing novel *EML4-ALK* fusion, crizotinib was administered orally. Regrettably, these cases did not achieve durable drug responses. For instance, following osimertinib failure and the detection of *EML4-ALK* variant 1 (V1), crizotinib was administrated as a posterior line therapy for a smoker patient, resulting in only a 4-month benefit, followed by brigatinib (Yan et al., 2020). Likewise, a transient response of crizotinib plus osimertinib was observed in a progressed lung adenocarcinoma patient previously treated with first-line gefitinib and second-line osimertinib (Hou et al., 2021).

Recently, the first-line treatment options for primary tumors with *ALK* rearrangement have expanded to include second- and third-generation ALK-TKIs (Peters et al., 2017; Camidge et al., 2018; Horn et al., 2021; Solomon et al., 2023). Consequently, the therapeutic approach for osimertinib-resistant patients with acquired *EML4-ALK* fusion is no longer limited to crizotinib. Among these alternatives, alectinib has been administrated to several patients; however, it exhibited clinical activities for less than 5 months (von Buttlar et al., 2021; Wang et al., 2023). Despite the low frequency of acquired *EML4-ALK* fusion in osimertinib-resistant cases, the significant number of patients with *EGFR* mutations undergo osimertinib treatment, along with the unfavorable clinical outcomes associated with subsequent crizotinib or alectinib treatment, underscores the necessity for further exploration of more effective treatment strategies to improve clinical outcomes.

Here, we report a case of resectable NSCLC harboring complex *EGFR* L858R/G719S, and *BRAF* V600E mutations. During adjuvant therapy, the patient experienced long-term benefits from the sequential monotherapy with erlotinib, osimertinib, and ensartinib. Notably, the third-line ensartinib demonstrated significantly improved efficacy and safety in this patient, who developed *EML4-ALK* fusion following resistance to osimertinib, providing valuable guidance for clinical decisions in patients management.

## 2 Case presentation

**FIGURE 1:**
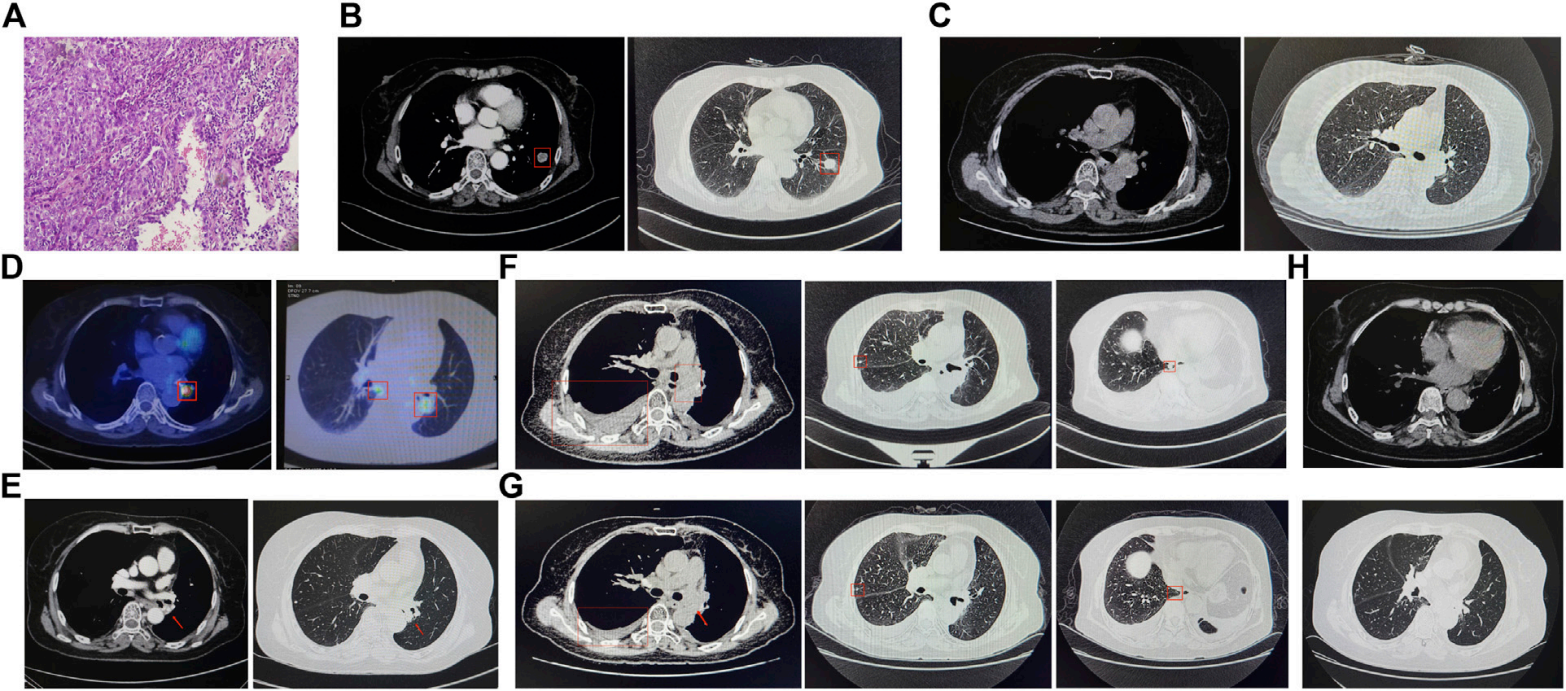
Sequential CT scan evaluation and histological examination analysis in this case report. **(A)** Pelvic puncture tissue biopsy. The CT scan image on June 2018 **(B)**, October 2018 **(C)**, April 2020 **(E)**, April 2021 **(F)**, June 2021 **(G)**, and July 2022 **(H)**. **(D)** The PET-CT scan on August.

On 5 June 2018, a 71-year-old Chinese female with no smoking history was admitted to our hospital presenting chronic cough and chest distress. Her medical history included hepatitis B, which had been in remission for 30 years, and a stable benign meningioma, with no treatment history. One month before admission, a fluorine 18 (18F)-labeled fluorodeoxyglucose (FDG) positron emission tomography (PET)/computed tomography (CT) identified a significant space-occupying lesion with irregular margins (1.7*1.8 cm) in her left lower lobe near the pleura. Additionally, three lymph nodes in mediastinum and left hilum showed signs of metastasis, with no distant metastases observed. Based on PET-CT scans, this patient was diagnosed with stage IIIA peripheral lung cancer with lymph node metastasis. A contrast-enhanced CT (CECT) scan further confirmed the presence of a nodule in the left lower lobe (Figure 1B). Small nodules in the right upper lung and adrenal glands were not detected by PET-CT, requiring further observation. Serum biochemistry tests revealed elevated carcinoembryonic antigen (CEA) and cytokeratin fragment 21–1 (Cyfra21-1), exceeding normal levels.

**FIGURE 2:**
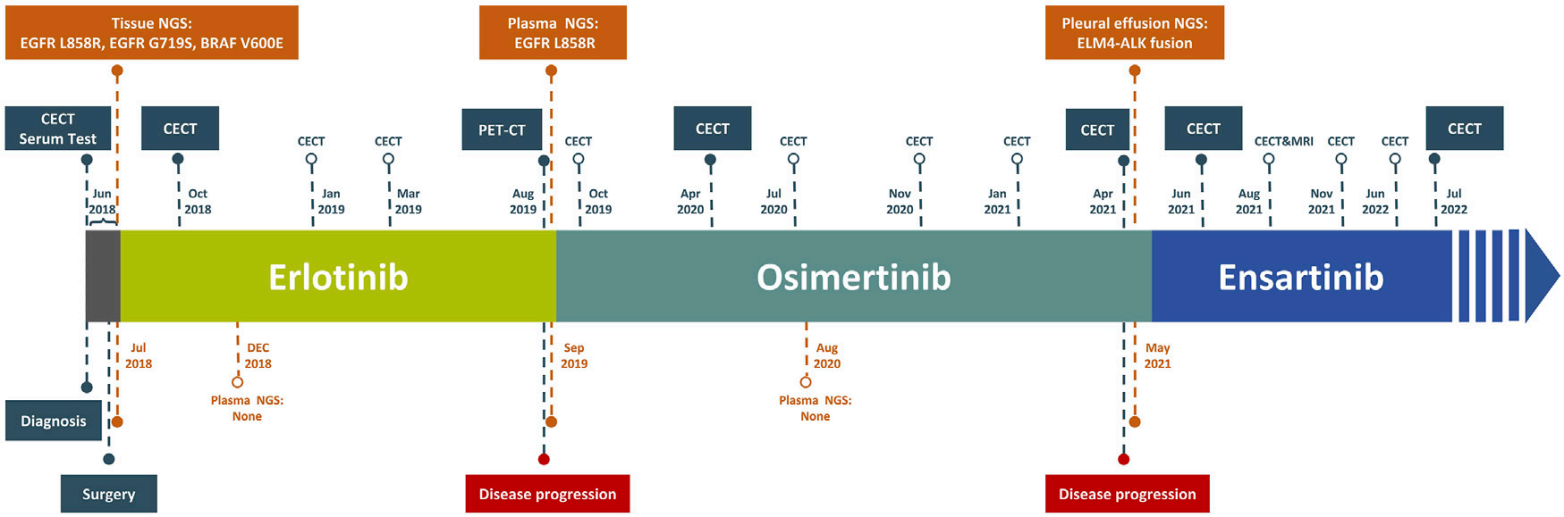
Timeline of events and detected mutations of this case report.

On 11 June 2018, with informed consent, this patient underwent a left lung lobotomy and complete mediastinal lymphadenectomy (Figure 2). Hematoxylin and eosin staining of the excised tumor mass confirmed a stage IIIA infiltrating lung adenocarcinoma of the solid predominant subtype (Figure 1A), with carcinoma metastasis in regional lymph nodes. Panel-based genomic DNA sequencing of tissue samples revealed compound somatic sensitive mutations (Repugene Technology, Hangzhou, People’s Republic of China), including *EGFR* L858R/G719S and *BRAF* V600E (Table 1). Given the high recurrence risk, she received adjuvant treatment with erlotinib. Four months after initiating targeted therapy, imaging showed no tumor lesions *in situ*, confirmed by the negative results of non-invasive liquid biopsy sequencing conducted 2 months later (Figure 1C; Table 1).

**TABLE 1:** Genomic alterations and molecular test of this case during treatment.

Event	Detection time	Biopsy samples	Mutations (MAFs %)
Surgery and Diagnosis	06/2018	FFPE	*EGFR* L858R (1.79%)
			*EGFR* G719S (2.05%)
			*BRAF* V600E (5.13%)
		FFPE	MSI: MSS
Erlotinib	12/2018	Blood	None
	09/2019 (Progression)	Blood	*EGFR* L858R (0.28%)
Osimertinib	07/2020	Blood	None
	04/2021 (Progression)	Pleural effusion	*EML4-ALK* (E6:A20) V3

After 13-month treatment of erlotinib, the PET-CT indicated intense FDG activity in nodules located in the inferior lobe of the left lung near the hilar, suggesting intrapulmonary lymph node metastases (Figure 1D). A subsequent plasma genotyping detected a recurring L858R mutation (Table 1). Osimertinib was tentatively employed as second-line treatment in September 2019, with significant tumor reduction observed 7 months later (Figure 1E). Ten months after initiating osimertinib, magnetic resonance imaging of the thoracolumbar vertebrae and routine CT scans revealed slight enlargement of pulmonary nodules, mildly increased bilateral pleural effusions, and potential bone metastases. In addition to continued osimertinib, pleural bevacizumab injections were started in August 2020 (10-month after initiating osimertinib) to control pleural effusions, administered monthly. After 19 months of osimertinib treatment, the patient developed new nodules and exhibited obviously increased bilateral pleural effusions, suggesting potential disease recurrence (Figure 1F). To determine the genetic alternations and subsequent therapeutic strategies, pleural effusion was sampled, and the supernatant was subjected to targeted panel sequencing in April 2021, which detected an acquired *EML4-ALK* variant 3 (E6:A20) mutation. As a result, ensartinib was administered in May 2021. One month later, a CECT assessment showed alleviated tumor lesion, decreased pleural effusions, and reduced nodules sizes (Figure 1G). Six months after initiating ensartinib, CT scan indicated further reduced pleural effusions and cleared nodules. At subsequent follow-up visits at 9- and 12-month post-ensartinib treatment, pleural effusions were persistently decreased. At the last follow-up in July 2022, CT scans showed resolved pleural effusions (Figure 1H), and serum marker levels remained within normal range. In addition to tolerable rash and minor abnormalities in liver and kidney function, no other side effects were observed over ensartinib treatment, indicating a favorable drug safety.

## 3 Discussion

Targeted therapy characterized by lower toxicity and physical burden is a standard treatment regimen for NSCLC patients carrying driver mutations with clinical significance. However, the inevitable development of resistance necessitates close monitoring through imaging and genetic testing. The development of sequential therapies for long-term treatment benefits from ongoing drug research and advancements in genetic testing technologies, especially non-invasive liquid biopsy. In this study, genotyping of resected tumor tissue from an elderly female patient diagnosed with lung adenocarcinoma reported the co-mutations of *EGFR* L858R/G719S and *BRAF* V600E. Dynamic monitoring via liquid biopsies identified L858R and *EML4-ALK* fusion as resistance mechanisms to erlotinib and osimertinib, respectively. The *EML4-ALK* fusion, an infrequent off-target alteration to osimertinib, was effectively targeted by ensartinib, achieving a 14-month disease-free survival, superior to crizotinib and aletinib in similar cases reported previously. This report of ensartinib’s clinical application in osimertinib-resistant patient provides valuable insights for decision-making in clinical management.

The detection of coexisting mutations of *EGFR* L858R/G719S and *BRAF* V600E in this patient posed a challenge in choosing the appropriate TKI for adjuvant therapy. BRAF, a key molecule in the EGFR/RAS downstream signaling pathway, is mutated in approximately 1.5%–5.5% NSCLC cases, with the predominant genotype being *BRAF* V600E (Jordan et al., 2017; Lin et al., 2019). Promising efficacy and safety have been demonstrated with BRAF inhibitors, such as dabrafenib (Planchard K. et al., 2016), vemurafenib (Subbiah et al., 2019), and the combination of dabrafenib plus trametinib (Planchard B. et al., 2016; Planchard et al., 2017; Ettinger et al., 2021), in clinical trials involving advanced *BRAF* V600E mutant NSCLC. The development of acquired *BRAF* V600E has been reported to render tumor insensitivity to EGFR-TKIs treatment (Ohashi et al., 2012; Ricordel et al., 2018; Westover et al., 2018), indicating potential clinical benefits of *EGFR* and *BARF* co-inhibition in cases with dual mutations. Indeed, an increasing number of cases have reported that advanced *EGFR*-mutant NSCLC patients that developed concomitant *BRAF* mutations following acquired resistance to EGFR-TKI treatment exhibit durable responses and good tolerance to combined EGFR and BRAF inhibitors treatment (Aboubakar Nana and Ocak, 2021; Ribeiro et al., 2021; Schaufler et al., 2021). For instance, a male patient with lung adenocarcinoma underwent genetic testing after osimertinib treatment failure, which revealed co-mutations of *EGFR* T790M/19del and *BRAF* V600E. Consequently, he switched to a combination therapy of debrafenib, trametinib, and osimertinib, that resulted in a complete remission (Meng et al., 2020). Another female patient who developed resistance to osimertinib was found to carry *EGFR* 19del plus *BRAF* V600E mutations, received debrafenib, trametinib, and osimertinib, and achieved a stable disease within 6-month of treatment (Zeng et al., 2021). These findings indicate that, in our case, introducing BRAF inhibitors could help improve survival benefits when combined with erlotinib. However, there is a lack of comprehensive clinical trials on this combination therapy, and the available clinical data are limited, particularly in cases with primary *BRAF* and *EGFR* mutations. Given that the resistance mechanisms of debrafenib, trametinib, and EGFR-TKI remained unclear which may limit subsequent therapeutic options, this patient opted for erlotinib monotherapy. A clinical response of 14 months confirmed that erlotinib is an effective therapy.

At the end of erlotinib treatment, plasma genotyping was conducted to guide the switch in therapeutic strategy due to the lack of tumor tissues, and this testing confirmed the presence of *EGFR* L858R mutation. According to a diagnostic analysis of 216 advanced NSCLC cases, liquid biopsies have shown a 96.5% specificity in detecting L858R (Schrock et al., 2018), suggesting the reliability of this result for our patient. Osimertinib has demonstrated superior efficacy over first- and second-generation EGFR-TKIs in clinical trials of advanced NSCLC with L858R mutations. The FLAURA study, which focused on treatment-naive patients with L858R, found that those treated with osimertinib had a prolonged PFS than those receiving standard EGFR-TKI (gefitinib or erlotinib) (14.4 months vs. 9.5 months) (Soria et al., 2018). Another meta-analysis also reported the longest PFS in osimertinib-treated group among 12 treatment strategies (Zhao Y. et al., 2019). These findings suggest that osimertinib could provide a durable and safe response for this patient and we started osimertinib treatment with patient’s consent. During the course of treatment, despite minor radiographic signs of progression, we continued osimertinib as no driver mutations were detectable in the plasma genetic testing. However, 19 months after initiating osimertinib, an *EML4-ALK* fusion emerged, indicating a change in the tumor’s genetic landscape.

The resistance mechanisms of second-line osimertinib have been systematically documented, reporting that various oncogene fusion events, including *ALK* fusions, occur in less than 10% of cases (Fu et al., 2022). In instances where patients developed acquired *EML4-ALK* fusion after osimertinib, these typically co-occurred with *EGFR* mutations such as T790M, L858R, and 19del. Consequently, a combination of osimertinib plus ALK inhibitors is employed. However, most patients experienced tumor progression within 6 months when osimertinib is combined with crizotinib or alectinib (Batra et al., 2020; Hou et al., 2021; von Buttlar et al., 2021; Wang et al., 2023; Yan et al., 2020). For example, one patient showed disease progression on CT scans just 1 month after starting second-line treatment with osimertinib and crizotinib, and switching to brigatinib only maintained a stable disease response for 4 months (Yan et al., 2020). Ensartinib, a newly-marketed second-generation ALK inhibitor, has shown superior efficacy to crizotinib in first-line treatment for both systemic and intracranial disease. It has also proven effective in cases resistant to crizotinib or in overcoming alectinib-induced adverse events (Batra et al., 2020; Yang et al., 2020; Horn et al., 2021). In light of this, our patient received ensartinib and maintained a good quality of life over a 14-month follow-up, showing better outcomes compared to prior cases treated with crizotinib or brigatinib after osimertinib resistance. It has been reported that another NSCLC patient, who developed *EML4-ALK* fusion after becoming resistant to another third-generation EGFR-TKI almonertinib, received crizotinib combined with almonertinib but only had a stable disease status for 1 month (Ren et al., 2022). This is a far less effective case, though it is important to acknowledge that the varying effectiveness between ensartinib and other ALK inhibitors might be influenced by the presence of concurrent *EGFR* mutations, as well as patient-specific factors. The efficacy of ensartinib in osimertinib-resistant patients with co-existing *EGFR* and *ALK* mutations requires further exploration.

During the clinical diagnosis and management of this case, several limitations need to be addressed. First, the preoperative clinical diagnosis of this patient was solely based on CT scans without histopathological confirmation through tissue biopsy, which could have provided a more definitive diagnosis. Second, the clinically significant mutations identified by next-generation sequencing were not validated using other techniques such as Sanger sequencing and quantitative polymerase chain reaction, which could have strengthened the reliability of the results. Lastly, the genotyping conducted using blood or pleural effusion samples were not further confirmed in tumor tissues, raising the possibility of false-negative results.

## 4 Conclusion

This case report details a NSCLC patient with complex driver mutations, specifically, *EGFR* L858R/G719S and *BRAF* V600E, in the primary tumor. Treatment involved sequential monotherapies of erlotinib, osimertinib, and ensartinib, guided by non-invasive liquid biopsies. Of note, the emergence of an *EML4-ALK* rearrangement as a resistance mechanism to osimertinib treatment was effectively targeted by ensartinib, providing a targeted solution to this specific resistance mechanism. This strategy underscores the potential of tailored treatments based on evolving genetic profiles in managing complex NSCLC cases.

## Data Availability

The original contributions presented in the study are included in the article; further inquiries can be directed to the corresponding author.

## References

[B1] Aboubakar Nana F. Ocak S. (2021). Targeting BRAF activation as acquired resistance mechanism to EGFR tyrosine kinase inhibitors in EGFR-mutant non-small-cell lung cancer. Pharmaceutics 13 (9), 1478. 10.3390/pharmaceutics13091478 34575554 PMC8471192

[B2] Alexander M. Kim S. Y. Cheng H. (2020). Update 2020: management of non-small cell lung cancer. Lung 198 (6), 897–907. 10.1007/s00408-020-00407-5 33175991 PMC7656891

[B3] Batra U. Sharma M. Amrith B. P. Mehta A. Jain P. (2020). EML4-ALK fusion as a resistance mechanism to osimertinib and its successful management with osimertinib and alectinib: case report and review of the literature. Clin. Lung Cancer 21 (6), e597–e600. 10.1016/j.cllc.2020.05.016 32620470

[B4] Camidge D. R. Kim H. R. Ahn M. J. Yang J. C. Han J. Y. Lee J. S. (2018). Brigatinib versus crizotinib in ALK-positive non-small-cell lung cancer. N. Engl. J. Med. 379 (21), 2027–2039. 10.1056/NEJMoa1810171 30280657

[B6] Ettinger D. S. Wood D. E. Aisner D. L. Akerley W. Bauman J. R. Bharat A. (2021). NCCN guidelines insights: non-small cell lung cancer, version 2.2021. J. Natl. Compr. Canc Netw. 19 (3), 254–266. 10.6004/jnccn.2021.0013 33668021

[B7] Fu K. Xie F. Wang F. Fu L. (2022). Therapeutic strategies for EGFR-mutated non-small cell lung cancer patients with osimertinib resistance. J. Hematol. Oncol. 15 (1), 173. 10.1186/s13045-022-01391-4 36482474 PMC9733018

[B8] Gazdar A. F. (2009). Activating and resistance mutations of EGFR in non-small-cell lung cancer: role in clinical response to EGFR tyrosine kinase inhibitors. Oncogene 28 (Suppl 1), S24–S31. 10.1038/onc.2009.198 19680293 PMC2849651

[B9] Ho C. C. Liao W. Y. Lin C. A. Shih J. Y. Yu C. J. Yang J. C. (2017). Acquired BRAF V600E mutation as resistant mechanism after treatment with osimertinib. J. Thorac. Oncol. 12 (3), 567–572. 10.1016/j.jtho.2016.11.2231 27923714

[B10] Horn L. Wang Z. Wu G. Poddubskaya E. Mok T. Reck M. (2021a). Ensartinib vs crizotinib for patients with anaplastic lymphoma kinase-positive non-small cell lung cancer: a randomized clinical trial. JAMA Oncol. 7 (11), 1617–1625. 10.1001/jamaoncol.2021.3523 34473194 PMC8414368

[B11] Hou H. Sun D. Zhang C. Liu D. Zhang X. (2021). ALK rearrangements as mechanisms of acquired resistance to osimertinib in EGFR mutant non-small cell lung cancer. Thorac. Cancer 12 (6), 962–969. 10.1111/1759-7714.13817 33506568 PMC7952795

[B12] Huang L. Jiang S. Shi Y. (2020). Tyrosine kinase inhibitors for solid tumors in the past 20 years (2001-2020). J. Hematol. Oncol. 13 (1), 143. 10.1186/s13045-020-00977-0 33109256 PMC7590700

[B13] Jordan E. J. Kim H. R. Arcila M. E. Barron D. Chakravarty D. Gao J. (2017). Prospective comprehensive molecular characterization of lung adenocarcinomas for efficient patient matching to approved and emerging therapies. Cancer Discov. 7 (6), 596–609. 10.1158/2159-8290.CD-16-1337 28336552 PMC5482929

[B14] Leonetti A. Sharma S. Minari R. Perego P. Giovannetti E. Tiseo M. (2019). Resistance mechanisms to osimertinib in EGFR-mutated non-small cell lung cancer. Br. J. Cancer 121 (9), 725–737. 10.1038/s41416-019-0573-8 31564718 PMC6889286

[B15] Lin Q. Zhang H. Ding H. Qian J. Lizaso A. Lin J. (2019). The association between BRAF mutation class and clinical features in BRAF-mutant Chinese non-small cell lung cancer patients. J. Transl. Med. 17 (1), 298. 10.1186/s12967-019-2036-7 31470866 PMC6716889

[B16] McCusker M. G. Russo A. Scilla K. A. Mehra R. Rolfo C. (2019). How I treat ALK-positive non-small cell lung cancer. ESMO Open 4 (Suppl 2), e000524. 10.1136/esmoopen-2019-000524 31423342 PMC6677959

[B17] Meng P. Koopman B. Kok K. Ter Elst A. Schuuring E. van Kempen L. C. (2020). Combined osimertinib, dabrafenib and trametinib treatment for advanced non-small-cell lung cancer patients with an osimertinib-induced BRAF V600E mutation. Lung Cancer 146, 358–361. 10.1016/j.lungcan.2020.05.036 32534795

[B18] Minari R. Bordi P. La Monica S. Squadrilli A. Leonetti A. Bottarelli L. (2018). Concurrent acquired BRAF V600E mutation and MET amplification as resistance mechanism of first-line osimertinib treatment in a patient with EGFR-mutated NSCLC. J. Thorac. Oncol. 13 (6), e89–e91. 10.1016/j.jtho.2018.03.013 29596911

[B19] Offin M. Somwar R. Rekhtman N. Benayed R. Chang J. C. Plodkowski A. (2018). Acquired ALK and RET gene fusions as mechanisms of resistance to osimertinib in EGFR-mutant lung cancers. JCO Precis. Oncol. 2, 1–12. 10.1200/PO.18.00126 PMC6447364 30957057

[B20] Ohashi K. Sequist L. V. Arcila M. E. Moran T. Chmielecki J. Lin Y. L. (2012). Lung cancers with acquired resistance to EGFR inhibitors occasionally harbor BRAF gene mutations but lack mutations in KRAS, NRAS, or MEK1. Proc. Natl. Acad. Sci. U S A 109 (31), E2127–E2133. 10.1073/pnas.1203530109 22773810 PMC3411967

[B21] Passaro A. Janne P. A. Mok T. Peters S. (2021). Overcoming therapy resistance in EGFR-mutant lung cancer. Nat. Cancer 2 (4), 377–391. 10.1038/s43018-021-00195-8 35122001

[B22] Peters S. Camidge D. R. Shaw A. T. Gadgeel S. Ahn J. S. Kim D. W. (2017). Alectinib versus crizotinib in untreated ALK-positive non-small-cell lung cancer. N. Engl. J. Med. 377 (9), 829–838. 10.1056/NEJMoa1704795 28586279

[B23] Planchard D. Besse B. Groen H. J. M. Souquet P. J. Quoix E. Baik C. S. (2016). Dabrafenib plus trametinib in patients with previously treated BRAF(V600E)-mutant metastatic non-small cell lung cancer: an open-label, multicentre phase 2 trial. Lancet Oncol. 17 (7), 984–993. 10.1016/S1470-2045(16)30146-2 27283860 PMC4993103

[B24] Planchard D. Kim T. M. Mazieres J. Quoix E. Riely G. Barlesi F. (2016). Dabrafenib in patients with BRAF(V600E)-positive advanced non-small-cell lung cancer: a single-arm, multicentre, open-label, phase 2 trial. Lancet Oncol. 17 (5), 642–650. 10.1016/S1470-2045(16)00077-2 27080216 PMC5006181

[B25] Planchard D. Smit E. F. Groen H. J. M. Mazieres J. Besse B. Helland A. (2017). Dabrafenib plus trametinib in patients with previously untreated BRAF(V600E)-mutant metastatic non-small-cell lung cancer: an open-label, phase 2 trial. Lancet Oncol. 18 (10), 1307–1316. 10.1016/S1470-2045(17)30679-4 28919011

[B26] Ren K. H. Qin W. W. Wang Y. Peng J. C. Hu W. X. (2022). Detection of an EML4-ALK fusion mutation secondary to epidermal growth factor receptor-tyrosine kinase inhibitor (EGFR-TKI) therapy for lung cancer: a case report. Ann. Palliat. Med. 11 (7), 2503–2509. 10.21037/apm-22-744 35927783

[B27] Ribeiro M. Knebel F. H. Bettoni F. Saddi R. Sacardo K. P. Canedo F. (2021). Impressive response to dabrafenib, trametinib, and osimertinib in a metastatic EGFR-mutant/BRAF V600E lung adenocarcinoma patient. NPJ Precis. Oncol. 5 (1), 5. 10.1038/s41698-021-00149-4 33580193 PMC7880994

[B28] Ricordel C. Friboulet L. Facchinetti F. Soria J. C. (2018). Molecular mechanisms of acquired resistance to third-generation EGFR-TKIs in EGFR T790M-mutant lung cancer. Ann. Oncol. 29 (suppl_1), i28–i37. 10.1093/annonc/mdx705 29462256

[B29] Schaufler D. Ast D. F. Tumbrink H. L. Abedpour N. Maas L. Schwabe A. E. (2021). Clonal dynamics of BRAF-driven drug resistance in EGFR-mutant lung cancer. NPJ Precis. Oncol. 5 (1), 102. 10.1038/s41698-021-00241-9 34921211 PMC8683498

[B30] Schrock A. B. Zhu V. W. Hsieh W. S. Madison R. Creelan B. Silberberg J. (2018). Receptor tyrosine kinase fusions and BRAF kinase fusions are rare but actionable resistance mechanisms to EGFR tyrosine kinase inhibitors. J. Thorac. Oncol. 13 (9), 1312–1323. 10.1016/j.jtho.2018.05.027 29883838

[B31] Solomon B. J. Bauer T. M. Mok T. S. K. Liu G. Mazieres J. de Marinis F. (2023). Efficacy and safety of first-line lorlatinib versus crizotinib in patients with advanced, ALK-positive non-small-cell lung cancer: updated analysis of data from the phase 3, randomised, open-label CROWN study. Lancet Respir. Med. 11 (4), 354–366. 10.1016/S2213-2600(22)00437-4 36535300

[B32] Soria J. C. Ohe Y. Vansteenkiste J. Reungwetwattana T. Chewaskulyong B. Lee K. H. (2018). Osimertinib in untreated EGFR-mutated advanced non-small-cell lung cancer. N. Engl. J. Med. 378 (2), 113–125. 10.1056/NEJMoa1713137 29151359

[B33] Subbiah V. Gervais R. Riely G. Hollebecque A. Blay J. Y. Felip E. (2019). Efficacy of vemurafenib in patients with non-small-cell lung cancer with BRAF V600 mutation: an open-label, single-arm cohort of the histology-independent VE-BASKET study. JCO Precis. Oncol. 3, 1–9. 10.1200/PO.18.00266 PMC7446432 32914022

[B34] von Buttlar X. Reuss J. E. Liu S. V. Kim C. (2021). EML4-ALK rearrangement as a mechanism of resistance to osimertinib in metastatic lung adenocarcinoma: a case report. JTO Clin. Res. Rep. 2 (6), 100179. 10.1016/j.jtocrr.2021.100179 34590027 PMC8474351

[B35] Wang L.-S. Chen S.-Q. Zhong X. Jiao X. D. Liu K. Qin B. D. (2023). Acquired EML4-ALK fusion and EGFR C797S in cis mutation as resistance mechanisms to osimertinib in a non-small cell lung cancer patient with EGFR L858R/T790M. Anticancer Drugs 34 (10), 1146–1150. 10.1097/CAD.0000000000001489 36728908

[B43] Westover D. Zugazagoitia J. Cho B. C. Lovly C. M. Paz-Ares L. (2018). Mechanisms of acquired resistance to first- and second-generation EGFR tyrosine kinase inhibitors. Ann. Oncol. 1 (suppl_1), i10–i19. 10.1093/annonc/mdx703 PMC6454547 29462254

[B36] Wu J. Savooji J. Liu D. (2016). Second- and third-generation ALK inhibitors for non-small cell lung cancer. J. Hematol. Oncol. 9, 19. 10.1186/s13045-016-0251-8 26951079 PMC4782349

[B37] Xu H. Shen J. Xiang J. Li H. Li B. Zhang T. (2019). Characterization of acquired receptor tyrosine-kinase fusions as mechanisms of resistance to EGFR tyrosine-kinase inhibitors. Cancer Manag. Res. 11, 6343–6351. 10.2147/CMAR.S197337 31372039 PMC6628603

[B38] Yan Y. Jiang G. Ma W. Li T. Wang L. (2020). Emerging EML4-ALK Variant 5 as a Concurrent Resistance Mechanism to Osimertinib in a Patient With EGFR E19del/T790M NSCLC. Clin. Lung Cancer 21 (6), 562–567. 10.1016/j.cllc.2020.05.009 32622727

[B39] Yang Y. Zhou J. Zhou J. Feng J. Zhuang W. Chen J. (2020). Efficacy, safety, and biomarker analysis of ensartinib in crizotinib-resistant, ALK-positive non-small-cell lung cancer: a multicentre, phase 2 trial. Lancet Respir. Med. 8 (1), 45–53. 10.1016/S2213-2600(19)30252-8 31628085

[B40] Zeng R. Luo L. Sun X. Bao Z. Du W. Dai R. (2021). EGFR/BRAF/MEK co-inhibition for EGFR-mutated lung adenocarcinoma patients with an acquired BRAF(V600E) mutation: a case report and review of literature. Cancer Drug Resist 4 (4), 1019–1027. 10.20517/cdr.2021.98 35582379 PMC8992450

[B41] Zeng Z. Wang T. He J. Wang Y. (2022). ALK-R3HDM1 and EML4-ALK fusion as a mechanism of acquired resistance to gefitinib: a case report and literature review. Front. Oncol. 12, 1010084. 10.3389/fonc.2022.1010084 36387181 PMC9660230

[B42] Zhao Y L. J. Cai X. Pan Z. Liu J. Yin W. Chen H. (2019). Efficacy and safety of first line treatments for patients with advanced epidermal growth factor receptor mutated, non-small cell lung cancer: systematic review and network meta-analysis. BMJ 367, l5460. 10.1136/bmj.l5460 31591158 PMC6778694

